# The College of Nursing (Limited by Guarantee)

**Published:** 1920-11-20

**Authors:** 


### The College of Nursing
### (Limited by Guarantee).

MEMBERS are reminded of the Extraordinary General Meeting to be held on Saturday afternoon, November 20, at 3 P.M., at the Royal Society of Medicine, to confirm the Resolutions passed at the meeting of November 4, 1920.

### UNEMPLOYMENT INSURANCE ACT.

AT the close of the meeting of members of the College on Saturday, November 20, at the Royal Society of Medicine, an expert will explain the above Act as it affects the nursing profession.

Discussion will be invited. It is thought many members will wish to avail themselves of this opportunity of becoming familiar with the details and administration of the Act. The meeting will commence at 3.30 P.M.

### EDINBURGH CENTRE.

THE second annual general meeting of members took place on Wednesday, November 10, in the Nurses' Club, 8 Drumsheugh Gardens. Miss Gill, R.R.C., presided.

The Hon. Secretary read the report and statement of accounts for the year, the adoption of which was moved by Miss L. Bennett, seconded by Miss McKinnon, and carried unanimously by the meeting.

The President and other office-bearers were re-elected. Of the eight members of the Executive who, according to the constitution of the Centre, retire annually (in alphabetical order), five were re-elected and three decided not to stand for re-election, in order to give other nurses more frequent opportunity of attending committees and taking an active part in the work of the Centre.

The transaction of business was followed by a somewhat lengthy discussion on the question of commissioned rank for military and naval nursing services. After a vote had been taken one of the members moved that "The Edinburgh Centre of the College of Nursing, while expressing an opinion favourable to the granting of commissioned rank to the members of the military and naval nursing services, feels that it is a matter mainly concerning the regular services, and for the members of these services to decide." This resolution was carried unanimously, and evidently expressed the feeling of the meeting.

At the close of the meeting subscriptions for the ensuing year were taken. Any member who does not receive an official receipt within a week is requested to communicate with Miss Cathcart, The Elms, Whitehouse Loan. Through the kindness of Miss Ada M. Gordon, the members were afterwards entertained to tea in the restaurant of the Club.

### BIRMINGHAM AND THREE COUNTIES LOCAL CENTRE.

A LECTURE will be given by Mr. G. P. Mills, F.R.C.S., in the Lecture Theatre of the General Hospital on Tuesday, November 23, at 5.30. Subject, "Some Surgical Lessons from the War." Members are invited. Non-members (trained nurses) will be welcomed. Admission, 1s. each.

